# Remission of refractory lichen amyloidosis with baricitinib^☆^

**DOI:** 10.1016/j.abd.2026.501363

**Published:** 2026-05-09

**Authors:** Thiago Lenoir da Silva, Gleison Vieira Duarte

**Affiliations:** a Desenvolver Clínica Médica, Florianópolis, SC, Brazil; b Instituto Bahiano de Imunoterapia, Salvador, BA, Brazil

*Dear Editor,*

Lichen amyloidosis is the most frequent form of primary localized cutaneous amyloidosis, characterized by hyperkeratotic, pruritic, and lichenoid papules, resulting from the dermal deposition of amyloid material derived from keratin, without systemic involvement.^1^ Its etiopathogenesis involves the apoptosis of basal keratinocytes and the subsequent dermal deposition of cytokeratin fragments, frequently associated with the activation of the IL-31 pathway, responsible for intense pruritus.^1^ Treatment is quite challenging, with limited responses to topical corticosteroids, retinoids, immunosuppressants, and phototherapy.^1^

The present report describes a 22-year-old woman with pruritic lesions of five years' duration, distributed in the anterior region of her legs (Fig. 1). Histopathological examination revealed acanthosis, hypergranulosis, and compact hyperorthokeratosis. PAS and crystal violet staining revealed globular structures in the papillary dermis, and Congo red stain showed discreet marking, supporting the diagnosis of lichen amyloidosis. The patient had undergone multiple conventional therapies, including high-potency topical corticosteroid (clobetasol 0.05%), cyclosporine 300 mg for six months, acitretin 50 mg for one month, amitriptyline 50 mg (still in use), and antihistamines, without significant clinical improvement. The lesions persisted with intense pruritus, significantly impacting her quality of life.

**Fig. 1:**
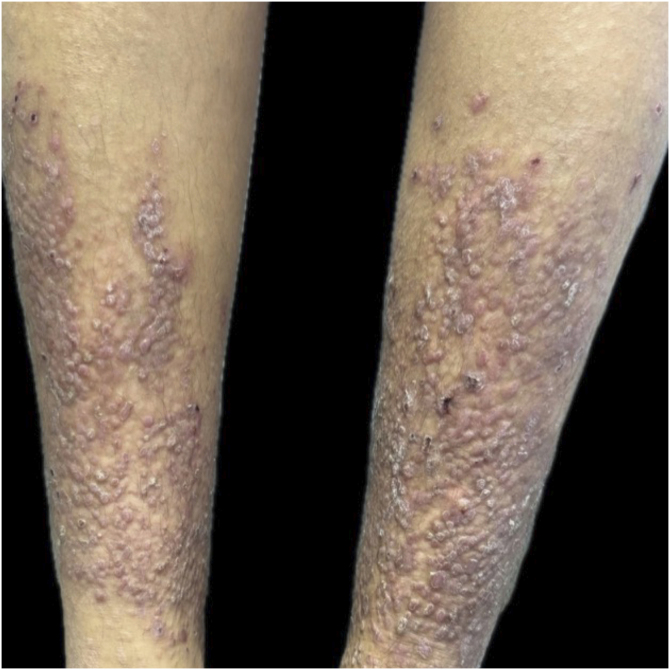
Erythematous and hyperkeratotic papules on the legs, before treatment.

Considering the refractory nature and emerging evidence on the role of JAK/STAT and IL-31 pathways in the pathophysiology of lichen amyloidosis,^1^, ^2^ baricitinib 4 mg/day was chosen, off-label, for the case. The drug was chosen for its safety profile in chronic inflammatory skin conditions and for previous reports of efficacy in cases of lichen amyloidosis associated with atopic dermatitis.^3^

The clinical response was rapid and significant. Pruritus ceased completely on the second day of treatment, and the lesions began to regress progressively with flattening, leaving residual hyperpigmentation. After 30 days, notable improvement in the texture and color of the lesions was observed (Fig. 2), progressing to almost complete resolution at 90 days (Fig. 3), when compared to the initial appearance. No adverse events were recorded during the three-month follow-up.

**Fig. 2:**
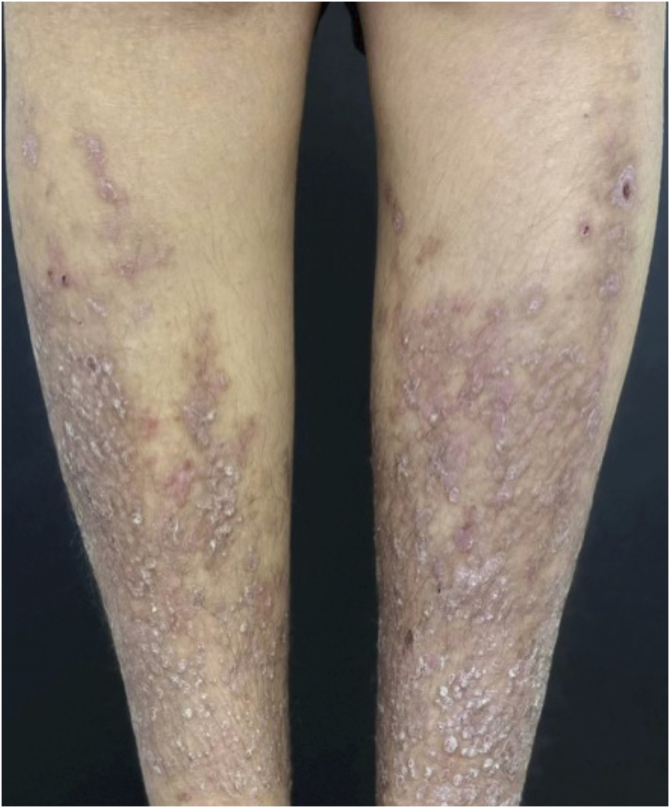
After 30 days of baricitinib 4 mg/day: Papules begin to resolve.

**Fig. 3:**
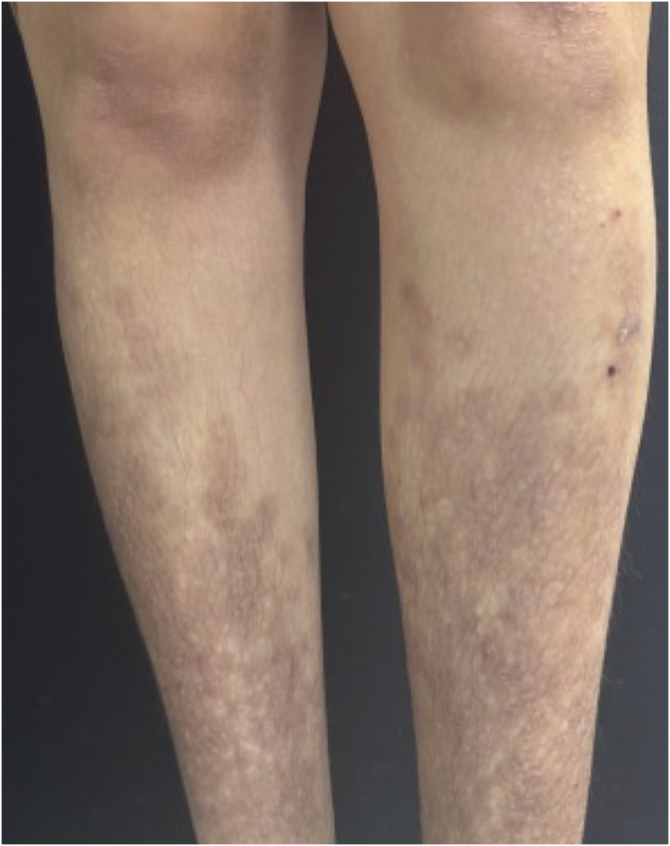
After 90 days of baricitinib 4 mg/day: almost complete resolution of the lesions, with residual hyperpigmentation.

Recent studies reinforce that lichen amyloidosis is frequently associated with alterations in signaling of the JAK-STAT pathways, especially JAK1 and JAK2, implicated in IL-31 activation and the pruritus-lesion-amyloid deposition cycle.^2^, ^3^ This pathophysiological basis explains the effectiveness of JAK inhibitors in controlling both pruritus and persistent dermal inflammation. Several reports support this rationale: the use of dupilumab has shown benefit in refractory cases of lichen amyloidosis, including when there is co-occurrence with atopic dermatitis;^4^, ^5^, ^6^ nemolizumab, an IL-31RA blocker, promoted complete remission after two years of use^2^; abrocitinib, a selective JAK1 inhibitor, resulted in marked clinical improvement and reduction of the EASI score in patients with lichen amyloidosis associated with atopic dermatitis^7^; and upadacitinib, another JAK1 inhibitor, demonstrated rapid and sustained responses in isolated cases of resistant lichen amyloidosis.^6^, ^8^

The present case corroborates these observations, demonstrating the effectiveness of baricitinib, a selective JAK1/JAK2 inhibitor, in the complete control of pruritus and clinical regression of lesions. Early improvement and the absence of adverse effects reinforce the potential of this class as a promising therapeutic alternative for refractory forms of lichen amyloidosis, especially when classic immunosuppressants and retinoids fail.

In conclusion, baricitinib proved to be an effective and safe option in the management of refractory lichen amyloidosis, with rapid resolution of pruritus and significant improvement of lesions in the short term. This report contributes to expanding the evidence on the use of JAK inhibitors in primary cutaneous amyloidosis, highlighting the need for controlled studies that consolidate their therapeutic role.

## Authors’ contributions

Thiago Lenoir da Silva: Design and planning of the study; drafting and editing of the manuscript; critical review of intellectual content; approval of the final version of the manuscript.

Gleison Vieira Duarte: Design and planning of the study; drafting and editing of the manuscript; critical review of intellectual content; approval of the final version of the manuscript.

## Financial support

None declared.

## Research data availability

Does not apply.

## Conflicts of interest

Thiago Lenoir da Silva declares potential conflicts of interest related to scientific, educational, or consulting activities with the following pharmaceutical companies: AbbVie, Sun Pharma, and Sanofi.

Gleison Duarte Vieira declares potential conflicts of interest related to scientific, educational, consulting, or research activities with the following pharmaceutical companies: AbbVie, Eli Lilly, Celldex, Amgen, Johnson & Johnson (J&J), Novartis, Galderma, LEO Pharma, UCB, Pfizer, Sun Pharma, and Sanofi.
